# How Uncertain is the Survival Extrapolation? A Study of the Impact of Different Parametric Survival Models on Extrapolated Uncertainty About Hazard Functions, Lifetime Mean Survival and Cost Effectiveness

**DOI:** 10.1007/s40273-019-00853-x

**Published:** 2019-11-25

**Authors:** Ben Kearns, John Stevens, Shijie Ren, Alan Brennan

**Affiliations:** School of Health and Related Research (ScHARR), University of Sheffield, Regent Court, 30 Regent Street, Sheffield, S1 4DA UK

## Abstract

**Background and Objective:**

The extrapolation of estimated hazard functions can be an important part of cost-effectiveness analyses. Given limited follow-up time in the sample data, it may be expected that the uncertainty in estimates of hazards increases the further into the future they are extrapolated. The objective of this study was to illustrate how the choice of parametric survival model impacts on estimates of uncertainty about extrapolated hazard functions and lifetime mean survival.

**Methods:**

We examined seven commonly used parametric survival models and described analytical expressions and approximation methods (delta and multivariate normal) for estimating uncertainty. We illustrate the multivariate normal method using case studies based on four representative hypothetical datasets reflecting hazard functions commonly encountered in clinical practice (constant, increasing, decreasing, or unimodal), along with a hypothetical cost-effectiveness analysis.

**Results:**

Depending on the survival model chosen, the uncertainty in extrapolated hazard functions could be constant, increasing or decreasing over time for the case studies. Estimates of uncertainty in mean survival showed a large variation (up to sevenfold) for each case study. The magnitude of uncertainty in estimates of cost effectiveness, as measured using the incremental cost per quality-adjusted life-year gained, varied threefold across plausible models. Differences in estimates of uncertainty were observed even when models provided near-identical point estimates.

**Conclusions:**

Survival model choice can have a significant impact on estimates of uncertainty of extrapolated hazard functions, mean survival and cost effectiveness, even when point estimates were similar. We provide good practice recommendations for analysts and decision makers, emphasizing the importance of considering the plausibility of estimates of uncertainty in the extrapolated period as a complementary part of the model selection process.

**Electronic supplementary material:**

The online version of this article (10.1007/s40273-019-00853-x) contains supplementary material, which is available to authorized users.

## Key Points for Decision Makers

**Table:** 

Guidance is available on choosing between parametric survival models used in a cost-effectiveness analysis. However, this does not consider the impact of model choice on uncertainty in extrapolated hazard functions and lifetime mean survival. Intuitively, we might expect that this uncertainty increases the further into the future we extrapolate.
We illustrate, using seven commonly applied parametric survival models and four hypothetical datasets, that the choice of survival model can have a marked impact on resulting estimates of uncertainty about the hazard function, lifetime mean survival and cost effectiveness. Estimates of uncertainty about extrapolated hazard functions could increase, decrease or be constant depending on the model used.
We provide recommendations on how the clinical plausibility of estimates of uncertainty about hazard functions and estimates of cost effectiveness should be used as part of the model selection process.

## Introduction

Estimates of lifetime mean survival are often a key component of cost-effectiveness analyses, as they typically quantify the benefits of new treatments. Cost-effectiveness analyses play an important role in reimbursement decisions [1]. Clinical trials typically have a shorter follow-up period then the time horizon required in a cost-effectiveness analysis. Hence, extrapolation of hazard functions is often required to estimate lifetime mean survival. This may be achieved by fitting commonly applied parametric survival models (as described in Sect. 2.1) to sample data. The National Institute for Health and Care Excellence Decision Support Unit Technical Support Document 14 describes different parametric survival models and suggestions for how to choose between them, highlighting the importance of considering uncertainty [2].

Extrapolation introduces additional uncertainty that does not occur for within-sample prediction. This is due to the absence of data to calibrate model estimates or validate their plausibility. For example, an exponential distribution may provide an adequate fit to the observed data. By definition, the suitability of the exponential model for the extrapolated period cannot be assessed from the observed data. External evidence, such as clinical opinion, may be used to support the plausibility of extrapolated estimates. However, even if the exponential distribution is deemed suitable, there remains uncertainty that the model parameter estimated from the observed data will be the same in the future. Hence, there is extrapolation uncertainty in both the suitability of the chosen model and the suitability of the estimated parameters. As such, there is often an expectation amongst analysts and decision makers that uncertainty about estimates of hazard functions (as quantified by their variance) should increase over the extrapolation period. The effect of this extrapolation uncertainty is recognised in the time-series literature, with extrapolations being associated with greater uncertainty than within-sample estimates [3, 4]. To our knowledge, there has been little consideration of whether the use of commonly applied parametric survival models adequately reflects extrapolation uncertainty.

Our study had two aims. The first was to illustrate the impact of model choice on estimates of uncertainty about extrapolated hazard functions, estimates of lifetime mean survival and estimates of cost effectiveness. The second aim was to raise awareness of this impact when producing and critiquing survival models. We begin Sect. 2 by showing how to derive estimates of uncertainty of extrapolated hazard functions and the estimated lifetime mean survival using both analytical expressions and approximation methods (delta and multivariate normal approach) for when exact analytical solutions are not tractable. We then create four representative hypothetical datasets, reflecting hazard functions commonly encountered in clinical practice for use in case studies, to illustrate the impact of model choice on estimates of uncertainty. We used one of these datasets to perform a hypothetical cost-effectiveness analysis. Section 3 presents the results of the case studies and the cost-effectiveness analysis. In Sect. 4, we provide recommendations on how to use the impact of survival model choice on estimates of uncertainty as part of the model selection process. We focus on extrapolating a single arm of a trial.

## Methods

### Commonly Applied Parametric Survival Models

For this study, we considered seven commonly applied parametric survival models: exponential, Weibull, Gompertz, gamma, log-logistic, log-normal and generalised gamma distributions. With the exception of the Gompertz distribution, these models all belong to the generalised F family of distributions [5, 6]. We originally also considered the generalised F model, but do not include it here, as the model estimation procedure did not always converge under the default settings [see Appendix 2 of the Electronic Supplementary Material (ESM) for more details]. The different survival models make different assumptions about their underlying hazard functions over time: an exponential distribution assumes a constant hazard; Weibull, Gompertz and gamma distributions allow for monotonically increasing or decreasing hazards over time; log-normal and log-logistic distributions allow the hazard function to be unimodal (also monotonically decreasing for the log-logistic) [6]. The generalised gamma distribution is the most flexible of the commonly applied models. It can model hazards that are constant, monotonic (increasing or decreasing), bathtub or arc shaped [7].

Table 1 describes the characteristics of seven commonly used survival models, including the survival function $S\left( t \right)$, hazard function $h\left( t \right)$ and cumulative hazard function $H\left( t \right)$. These three functions are all related via the equation:

1 $$H\left( t \right) = \mathop \int \nolimits_{0}^{t} h\left( u \right) {\text{d}}u = - \ln \left( {S\left( t \right)} \right).$$

**Table 1:** Overview of seven commonly used survival models and their characteristics (*t* ≥ 0)

Model (parameters)	Survival function $S\left( t \right)$	Cumulative hazard function $H\left( t \right)$	Hazard function $h\left( t \right)$	Possible shapes of the hazard function
Exponential $\lambda > 0$	${\text{e}}\left( { - \lambda t} \right)$	$\lambda t$	$\lambda$	Constant
Weibull $\lambda > 0, \;\gamma > 0$	${\text{e}}\left( { - \lambda t^{\gamma } } \right)$	$\lambda t^{\gamma }$	$\lambda \gamma t^{\gamma - 1}$	Constant Increasing monotonically Decreasing monotonically
Lognormal $$\mu \in \left( { - \infty ,\infty } \right),$$$$\sigma > 0$$	$$1 - \varPhi \left( {\frac{\log t - \mu }{\sigma }} \right)$$	$$- \ln \left( {1 - \varPhi \left( {\frac{\log \left( t \right) - \mu }{\sigma }} \right)} \right)$$	$$\frac{1}{{\sigma \sqrt {2\pi } }}t^{ - 1} {\text{e}}\left\{ { - \frac{{(\log t - \mu )^{2} }}{{2\sigma^{2} }}} \right\}/S\left( t \right)$$	Increasing then decreasing
Log-logistic$\alpha > 0, \;\beta > 0$	$\frac{1}{{1 + \left( {\alpha t} \right)^{\beta } }}$	$- \ln \left( {\frac{1}{{1 + \left( {\alpha t} \right)^{\beta } }}} \right)$	$\frac{{\alpha \beta \left( {\alpha t} \right)^{\beta - 1} }}{{1 + \left( {\alpha t} \right)^{\beta } }}$	Decreasing monotonically Increasing then decreasing
Gamma $\lambda > 0, \;\beta > 0$	$1 - \varGamma \left[ {\lambda^{ - 2} {\text{e}}^{ - \beta } t;\,\lambda^{ - 2} } \right]$	$- \ln \left( {S\left( t \right)} \right)$	$\frac{{\beta^{\lambda } t^{\lambda - 1} {\text{e}}^{ - \beta t} }}{\varGamma \left( \lambda \right)S\left( t \right)}$	Constant Increasing monotonically Decreasing monotonically
Generalised gamma $\lambda \in \left( { - \infty ,\infty } \right), \beta > 0, \;\sigma > 0$	$$\left\{ {\begin{array}{*{20}c} {1 - \varGamma \left[ {\lambda^{ - 2} \left( {{\text{e}}^{ - \beta } t} \right)^{{\frac{\lambda }{\sigma }}} ;\,\lambda^{ - 2} } \right] \quad {\text{if}}\, \lambda > 0} \\ {\varGamma \left[ {\lambda^{ - 2} \left( {{\text{e}}^{ - \beta } t} \right)^{{\frac{\lambda }{\sigma }}} ;\,\lambda^{ - 2} } \right] \quad {\text{if}}\, \lambda < 0} \\ \end{array} } \right.$$	$- \ln \left( {S\left( t \right)} \right)$	$\frac{\left| \lambda \right|}{{\sigma t\varGamma \left( {\lambda^{ - 2} } \right)}}\alpha \lambda^{ - 2} {\text{e}}^{ - \alpha } /S\left( t \right)$, where $\alpha = \lambda^{ - 2} \left( {{\text{e}}^{ - \beta } t} \right)^{\lambda /\sigma }$	Constant Increasing monotonically Decreasing monotonically Bathtub Arc-shaped
Gompertz $\lambda > 0, \theta \in \left( { - \infty ,\infty } \right)$	${\text{e}}\left\{ { - \frac{\lambda }{\theta }\left( {{\text{e}}^{\theta t} - 1} \right)} \right\}$	$\frac{\lambda }{\theta }\left( {{\text{e}}^{\theta t} - 1} \right)$	$\lambda {\text{e}}^{\theta t}$	Constant Increasing monotonically Decreasing monotonically

$\varPhi$ is the cumulative standard normal distribution; $\varGamma \left( {t;\,\lambda } \right) = \mathop \int \nolimits_{0}^{t} \frac{{x^{\lambda - 1} {\text{e}}^{ - x} {\text{d}}x}}{\varGamma \left( \lambda \right)}$ , and ${\text{e}}$ denotes the exponential function. Allowing $\theta < 0$ for the Gompertz implies that the survival function will never equal 0

We focus on the hazard function because it provides insights into the natural history of a disease along with any time-varying responses to treatment [8]. We also consider the survival function because this is a clinically important statistic.

### Estimating Uncertainty About Hazard and Survival Estimates

In this section, we describe how to quantify the uncertainty in the hazard and survival functions and uncertainty in estimates such as mean survival time. For illustration, we take a frequentist perspective and estimate parameters using maximum likelihood. Ideally, exact analytic expressions of variance would be available for the estimates of interest (hazard and survival functions, and mean survival time). However, as these are estimates of non-linear functions of model parameters, approximation methods are required.Exact analytical expressions are available for the exponential model. The maximum likelihood estimate of the model parameter $\lambda$ is:

2 $$\widehat{\lambda } = \frac{{\sum \delta_{i} }}{{\sum t_{i} }} = \frac{{N_{\text{e}} }}{{\sum t_{i} }},$$

where the subscript *i* denotes an individual, $\delta_{i} = 1$ for an event and zero otherwise, $t_{i}$ represents the observed times and $N_{\text{e}}$ represents the number of events. As described in Collet [6], the variance of the estimated hazard function is the variance of the estimated model parameter $\widehat{\lambda },$ given by:

3 $${\text{Var}}\left( {\widehat{h\left( t \right)}} \right) = {\text{Var}}\left( {\widehat{\lambda }} \right) = \frac{{\sum \delta_{i} }}{{\left( {\sum t_{i} } \right)^{2} }} = \frac{{\left( {\widehat{\lambda }} \right)^{2} }}{{N_{\text{e}} }}.$$

From Eq. 3, the variance of the hazard function is constant with respect to time, which means that the uncertainty does not ‘fan out’ over time. Thus, for the exponential model, uncertainty about the hazard function depends only upon the sample data that are used to estimate $\lambda$ and does not depend on whether we are considering the observed or unobserved period.

Estimates of uncertainty about the exponential survival function can be derived from the hazard function by using the relationship in Eq. 1. For the exponential model, the estimate of mean survival $\widehat{\mu }$ is given by:

4 $$\widehat{\mu } = \frac{1}{{\widehat{\lambda }}} = \frac{{\sum t_{i} }}{{N_{\text{e}} }}.$$

A confidence interval for the estimated mean survival may be derived via the delta method:

5 $${\text{Var}}\left( {\widehat{\mu }} \right) \approx \frac{1}{{\left( {\widehat{\lambda }} \right)^{4} }}{\text{Var}}\left( {\widehat{\lambda }} \right) = \frac{1}{{\left( {\widehat{\lambda }} \right)^{2} N_{\text{e}} }}.$$

Exact analytical expressions of variance (for hazard and survival functions) are not available for the other six commonly used parametric survival models. Two different approximation methods are commonly used to estimate variances of a function: the delta method [9] and the multivariate normal method [10].

The delta method estimates the variance of a function based on a linear approximation of the function [6]. The delta method may be used whenever the derivative of a function can be calculated. This includes all of the commonly used parametric survival functions in Table 1. To illustrate its use, we use the delta method to estimate the variance of the hazard function for both the exponential and Weibull models in Appendix 1 of the ESM. For the exponential model, applying the delta method gives the same equation for variance in the hazard as Eq. 3.

The multivariate normal method assumes that the estimated model parameters $\widehat{\theta }$ follow a multivariate normal distribution: $N\left( {\widehat{\theta }, {\text{Var}}\left[ {\widehat{\theta }} \right]} \right)$, where ${\text{Var}}\left( {\widehat{\theta }} \right)$ is estimated during model fitting. For example, $\widehat{\theta } = \left( {\widehat{\lambda },\widehat{\gamma }} \right)$ for the Weibull model, and ${\text{Var}}\left( {\widehat{\theta }} \right)$ is the estimated variance-covariance matrix. Parameter samples are drawn from the normal distribution and used to generate sample estimates of both the hazard and survival functions using the formulas in Table 1. Variances and confidence intervals are then derived from these sample estimates. The multivariate normal method has been shown to provide similar estimates of uncertainty to the delta method [10]. Its main advantage over the delta method is that it is easier to implement as it avoids calculating derivatives.

The multivariate normal approximation is a Monte Carlo simulation-based method. If $B$ Monte Carlo parameter samples are drawn from $N\left( {\widehat{\theta }, {\text{Var}}\left[ {\widehat{\theta }} \right]} \right)$, with a single sample denoted as $\theta_{b}$ ($b = 1, \ldots ,B$), then the variance of a function of the parameters, ${\text{Var}}\left( {g\left( \theta \right)} \right)$, is approximated as:

6 $${\text{Var}}\left( {g\left( \theta \right)} \right) \approx \frac{1}{B}\mathop \sum \limits_{b = 1}^{B} \left[ {g\left( {\theta_{b} } \right) - g\left( {\widehat{\theta }} \right)} \right]^{2} .$$

As this is a simulation-based method, it is not possible to derive analytic expressions for specific models, as in the case of the delta method for the Weibull in Appendix 1 of the ESM. Both the delta method and the multivariate normal approximation are used in common statistical software; the former in STATA and the latter in the flexsurv package in R [11, 12].

### Case Study: Datasets

We created four representative datasets to illustrate the impact of model choice on uncertainty in the estimated hazard and survival functions and mean survival. We generated all four datasets to have a sample size of 400, and mean survival of 0.9 years. We generated a dataset with a maximum follow-up of 1 year; any individuals who had not experienced an event by then were censored at 1 year. We applied no other censoring when creating the datasets. Each dataset may be viewed as describing outcomes for a single arm of a clinical trial, and was designed to represent different common hazard patterns:

1. A constant hazard, based on 400 Monte Carlo samples from an exponential distribution.
2. A monotonically increasing hazard, based on 200 Monte Carlo samples from a Weibull distribution and 200 Monte Carlo samples from a gamma distribution.
3. A monotonically decreasing hazard, based on 200 Monte Carlo samples from a Weibull distribution and 200 Monte Carlo samples from a gamma distribution.
4. A unimodal hazard, based on 200 Monte Carlo samples from a log-logistic distribution and 200 Monte Carlo samples from a log-normal distribution.

For datasets 2–4, we used a mixture of distributions to avoid the dataset’s characteristics being driven by a single model.

Our intention was not to perform a simulation study. Simulation studies are useful tools for quantitatively evaluating the performance of statistical methods under certain scenarios [13]. In contrast, the aim of this study was to explore the qualitative behaviour of interval estimates arising from different survival models, and how these depend on model choice.

### Case Study: Model Fitting and Analysis

We analysed the datasets assuming no knowledge of the distributions from which they were generated. We followed standard modelling practice by producing visual summaries of the data as part of an exploratory data analysis [14, 15]. We used two approaches to visualise the empirical hazard function: (1) smooth estimates of the empirical hazard over time based on kernel density smoothing, and (2) unsmoothed estimates using piecewise time periods. We used the functions muhaz and pehaz from the muhaz package [16] in R to generate the smoothed and unsmoothed versions, respectively (the number of piecewise time periods was 25 based on default options). The advantage of examining both of these empirical estimates of the hazard function is that the smoothed estimates are expected to capture the underlying shape of the hazard function represented by the sample data, whilst the unsmoothed versions highlight the variability in the data.

We fitted each of the models in Table 1 to each of the four datasets using the flexsurv package in R [12]. We then used each of the seven models to extrapolate hazard and survival functions for a lifetime. We used the multivariate normal method (the default approach in the flexsurv package) to generate 95% confidence intervals for the estimated hazard and survival functions. We used visual goodness of fit to identify a candidate set of plausible extrapolation models. We calculated estimates of mean survival and the uncertainty in these estimates for the candidate models, as these are an important summary measure in cost-effectiveness analyses.

We also performed a hypothetical cost-effectiveness analysis. This used the increasing hazards dataset (to reflect the impact of ageing), and a two-state “well”, “dead” Markov model, with utility values of 1 and 0, respectively. We used hazard estimates from the candidate models to represent outcomes for a control treatment, assuming it would cost £100 every 2 weeks. We also assumed the intervention treatment would have a hazard ratio of 0.75 (applied directly to the hazard estimates) and cost an additional £100 every 2 weeks. We used a lifetime horizon of 10 years, with weekly cycles. The cost-effectiveness measure used was the incremental cost per quality-adjusted life-years gained. The probabilistic sensitivity analysis used 1000 samples.

## Results

**Fig. 1:**
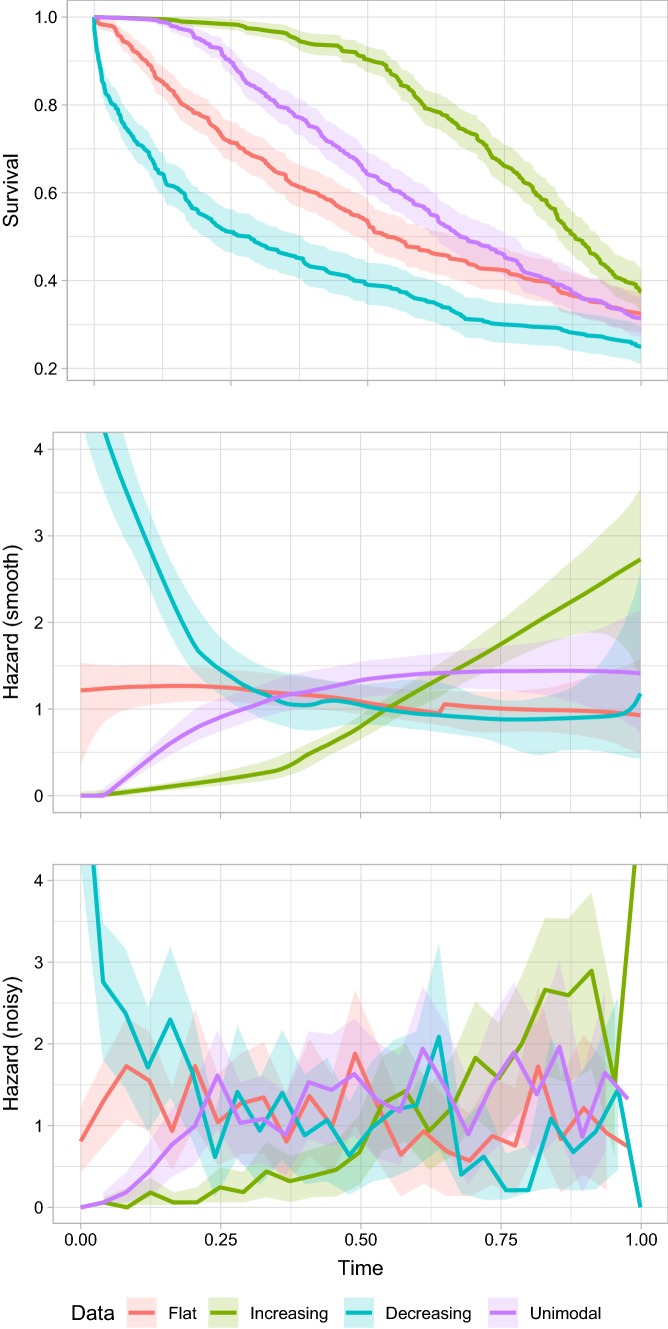
Visualisation of the Kaplan–Meier survival function estimate (with 95% confidence interval) and empirical hazard estimates in the observed 12-month period

Figure 1 provides the characteristics of the four representative datasets, showing the Kaplan–Meier survival function for each dataset, and the smooth and piecewise estimators of the hazard function. Figure 1 also includes 95% confidence intervals: for the survival functions these are based on Greenwood’s formula [6] and for the hazard estimates these are obtained via bootstrapping, as analytical formulae are not available. Figure 1 demonstrates that the characteristics of the datasets are as expected.

**Fig. 2:**
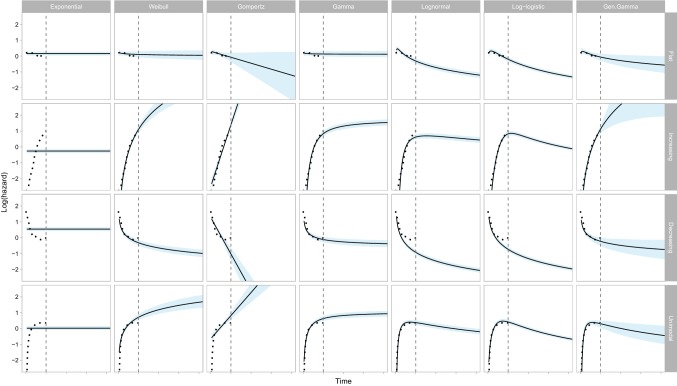
Visualisation of the estimated hazard (and 95% confidence interval) in the observed and extrapolated periods for seven commonly used statistical time-to-event models studied in four hypothetical datasets. The dotted line shows the observed (smoothed) hazard and the vertical dashed line denotes the end of the observed time period

Figure 2 provides the seven model-based estimates of the hazard function with 95% confidence intervals. As the hazard function is bounded below by zero, confidence intervals cannot fan out indefinitely. Instead, the logarithm of the hazard (which is not bounded) is displayed. Table 2 provides estimates for selected time periods. The exponential distribution assumes a constant hazard at all time-points. Hence, it only provides a good visual fit to the flat hazard dataset (see Fig. 2, first column). We also observed a poor visual fit for the Gompertz model for both the unimodal and decreasing hazard datasets. For the decreasing hazard dataset, we also observed a poor fit for the log-normal and log-logistic models.

**Table 2:** Estimates of the hazard and its standard error for seven commonly used statistical time-to-event models studies in four hypothetical datasets

Dataset and model	Time period (years)							
	0.5	1	1.5	2	2.5	3	3.5	4
Flat hazard								
Empirical (smooth)	1.09 (0.35)	0.93 (1.11)						
Exponential	1.16 (0.14)	1.16 (0.14)	1.16 (0.14)	1.16 (0.14)	1.16 (0.14)	1.16 (0.14)	1.16 (0.14)	1.16 (0.14)
Weibull	1.14 (0.16)	1.11 (0.21)	1.09 (0.24)	1.08 (0.27)	1.07 (0.28)	1.06 (0.30)	1.05 (0.31)	1.05 (0.33)
Gompertz	1.12 (0.14)	0.93 (0.27)	0.77 (0.38)	0.64 (0.47)	0.53 (0.53)	0.44 (0.58)	0.36 (0.61)	0.30 (0.64)
Gamma	1.15 (0.16)	1.14 (0.19)	1.13 (0.21)	1.13 (0.22)	1.13 (0.22)	1.13 (0.23)	1.13 (0.23)	1.12 (0.23)
Log-logistic	1.14 (0.17)	0.82 (0.13)	0.62 (0.09)	0.50 (0.07)	0.42 (0.06)	0.36 (0.05)	0.31 (0.04)	0.28 (0.04)
Log-normal	1.03 (0.14)	0.73 (0.11)	0.58 (0.09)	0.49 (0.08)	0.42 (0.07)	0.37 (0.06)	0.34 (0.06)	0.31 (0.05)
Gen. Gamma	1.12 (0.16)	0.94 (0.21)	0.83 (0.26)	0.75 (0.28)	0.69 (0.29)	0.64 (0.30)	0.61 (0.31)	0.57 (0.32)
Increasing hazard								
Empirical (smooth)	0.80 (0.27)	2.73 (1.95)						
Exponential	0.77 (0.09)	0.77 (0.09)	0.77 (0.09)	0.77 (0.09)	0.77 (0.09)	0.77 (0.09)	0.77 (0.09)	0.77 (0.09)
Weibull	0.72 (0.12)	3.01 (0.58)	6.94 (2.25)	12.53 (5.33)	19.83 (9.98)	28.86 (16.44)	39.63 (25.37)	52.15 (36.08)
Gompertz	0.58 (0.10)	4.02 (0.84)	28.02 (12.87)	195.31 (144.58)	1361.3 (1,497.7)	9488 (14,576)	66,130 (137,480)	460,913 (1,274,871)
Gamma	0.85 (0.13)	2.30 (0.38)	3.18 (0.57)	3.71 (0.71)	4.06 (0.80)	4.30 (0.85)	4.48 (0.89)	4.62 (0.92)
Log-logistic	0.79 (0.13)	2.28 (0.34)	2.18 (0.30)	1.78 (0.22)	1.46 (0.17)	1.23 (0.14)	1.06 (0.12)	0.93 (0.10)
Log-normal	0.96 (0.14)	1.86 (0.29)	2.01 (0.36)	1.96 (0.36)	1.86 (0.35)	1.76 (0.33)	1.66 (0.32)	1.57 (0.30)
Gen. Gamma	0.72 (0.13)	3.07 (0.78)	7.51 (7.56)	14.4 (35.33)	23.97 (113.17)	36.43 (260.86)	51.93 (330.16)	70.63 (370.34)
Decreasing hazard								
Empirical (smooth)	1.05 (0.45)	1.18 (2.10)						
Exponential	1.72 (0.20)	1.72 (0.20)	1.72 (0.20)	1.72 (0.20)	1.72 (0.20)	1.72 (0.20)	1.72 (0.20)	1.72 (0.20)
Weibull	1.01 (0.15)	0.73 (0.13)	0.60 (0.12)	0.53 (0.11)	0.47 (0.10)	0.43 (0.10)	0.4 (0.09)	0.38 (0.09)
Gompertz	1.14 (0.19)	0.36 (0.15)	0.11 (0.08)	0.04 (0.04)	0.01 (0.02)	< 0.01 (0.01)	< 0.01 (< 0.01)	< 0.01 (< 0.01)
Gamma	1.08 (0.16)	0.89 (0.15)	0.81 (0.15)	0.77 (0.15)	0.74 (0.15)	0.72 (0.15)	0.7 (0.15)	0.69 (0.15)
Log-logistic	0.82 (0.12)	0.48 (0.07)	0.34 (0.05)	0.27 (0.04)	0.22 (0.03)	0.19 (0.02)	0.16 (0.02)	0.15 (0.02)
Log-normal	0.68 (0.09)	0.40 (0.06)	0.29 (0.04)	0.23 (0.03)	0.19 (0.03)	0.17 (0.02)	0.15 (0.02)	0.13 (0.02)
Gen. Gamma	1.04 (0.17)	0.80 (0.20)	0.68 (0.23)	0.62 (0.25)	0.57 (0.27)	0.53 (0.29)	0.51 (0.30)	0.48 (0.31)
Unimodal hazard								
Empirical (smooth)	1.33 (0.42)	1.41 (1.41)						
Exponential	1.03 (0.12)	1.03 (0.12)	1.03 (0.12)	1.03 (0.12)	1.03 (0.12)	1.03 (0.12)	1.03 (0.12)	1.03 (0.12)
Weibull	1.27 (0.16)	2.04 (0.38)	2.69 (0.67)	3.28 (0.96)	3.82 (1.28)	4.33 (1.60)	4.81 (1.92)	5.27 (2.24)
Gompertz	1.09 (0.13)	2.26 (0.52)	4.69 (1.94)	9.73 (6.12)	20.17 (17.75)	41.81 (48.32)	86.68 (128.88)	179.69 (337.89)
Gamma	1.36 (0.18)	1.88 (0.31)	2.13 (0.38)	2.28 (0.43)	2.38 (0.46)	2.45 (0.48)	2.50 (0.49)	2.54 (0.50)
Log-logistic	1.47 (0.21)	1.52 (0.22)	1.23 (0.17)	0.99 (0.13)	0.82 (0.10)	0.70 (0.08)	0.60 (0.07)	0.53 (0.06)
Log-normal	1.45 (0.19)	1.46 (0.23)	1.31 (0.22)	1.18 (0.20)	1.07 (0.19)	0.98 (0.17)	0.90 (0.16)	0.84 (0.15)
Gen. Gamma	1.46 (0.19)	1.36 (0.31)	1.16 (0.39)	1.01 (0.43)	0.89 (0.44)	0.80 (0.44)	0.72 (0.44)	0.66 (0.43)

*Gen. Gamma* generalised gamma

Of the remaining candidate models, the width of confidence intervals always decreased during the extrapolated phase for the log-logistic model. For all other models, there was an increase in the interval width, although this was generally slight for both the log-normal and the gamma distributions. For the flat hazard dataset, all seven models provide visually good fits to the observed data. The exponential, Weibull and Gamma models all extrapolate a (near) constant hazard, whilst the remaining models extrapolate a decreasing hazard. If external evidence or clinical opinion was available to inform the likely long-term behaviour of the hazard (constant or decreasing), this could be used to reduce the set of candidate models to at most three or four models. The choice between the remaining models may then be informed by the behaviour of the extrapolated hazard. For example, of the constant hazard extrapolations, estimates of uncertainty from the Weibull model are the closest to reflecting increasing uncertainty over time. If it is not possible to choose between constant and decreasing hazard models, then the Gompertz model may be preferred as the only model for which the uncertainty in extrapolations includes the possibility of both constant and decreasing hazards. Similar remarks hold for the other datasets. For example, given the variety in the plausible long-term extrapolations arising from the increasing hazards dataset, all of the models appear to underestimate extrapolation uncertainty, with the potential exception of the generalised gamma.

**Fig. 3:**
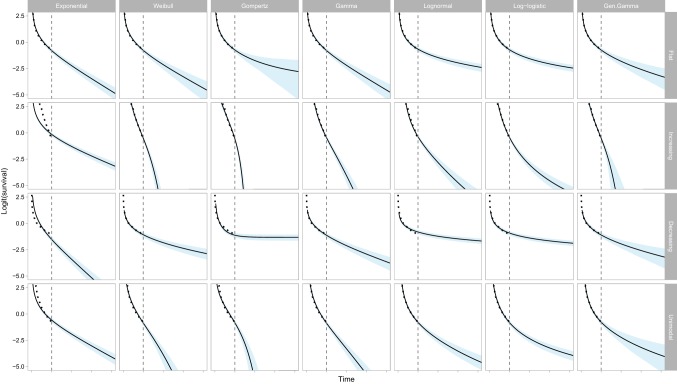
Visualisation of the estimated survival (and 95% confidence interval) in the observed and extrapolated periods for seven commonly used statistical time-to-event models studied in four hypothetical datasets. The dotted line indicates the observed survival and the vertical dashed line denotes the end of the observed time period

Figure 3 provides graphs of the estimated survival functions over time and 95% confidence intervals on the logit scale to make them unbounded. It is easier to interpret the long-term behaviour of the models from the hazard plots (for example, from the survival plots, it is not clear which models are extrapolating a constant hazard for the flat hazard dataset). The visual lack of fit of the models is also generally easier to interpret from the hazard plots. Note that when using the Gompertz distribution with a decreasing hazard, the extrapolated survival function will not reach zero (that is, it estimates that a proportion of individuals will never die).

**Fig. 4:**
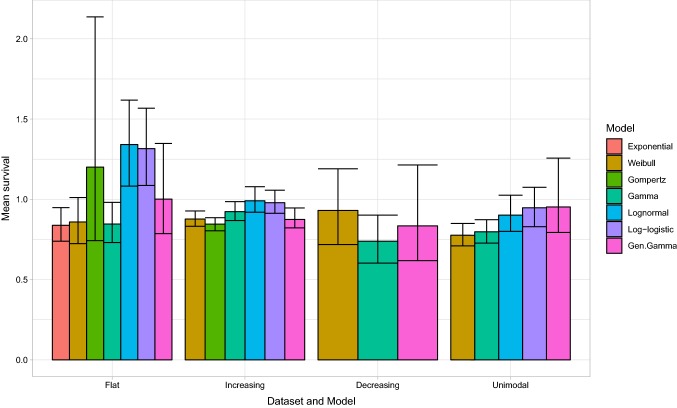
Estimates of lifetime mean survival and uncertainty (95% confidence interval) for seven commonly used statistical time-to-event models studied in four hypothetical datasets

Figure 4 displays estimates of lifetime mean survival for the candidate models. The results demonstrate that model choice influences not only the point estimates of mean survival but also the uncertainty about these estimates. For the flat hazard dataset, the estimated standard error in the mean survival arising from the Gompertz model (0.36) is almost seven times larger than the estimate arising from the exponential model (0.05), and about three times larger than the estimates from the log-logistic and log-normal models (0.12 and 0.13, respectively), which provide similar point estimates of mean survival. For the increasing hazard dataset, this difference in the estimate of uncertainty is reversed, with estimated standard errors from the log-logistic and log-normal models (both 0.04) being almost twice those from the Gompertz model (0.02).

Appendix 2 of the ESM provides the summary cost-effectiveness results. There was substantial variation in the estimates of the mean incremental cost-effectiveness ratios from the six candidate models (from £18,500 to £29,600, both per quality-adjusted life-year) and their associated uncertainty, with the widths of the confidence intervals ranging over threefold, from £4400 to £14,500. Even when models provided near-identical point estimates (£29,500 and £29,600 for the Weibull and generalised gamma, respectively), there remained large variation in the width of confidence intervals (£8400 and £14,500 respectively). For any given model, the expected value of information, which quantifies how much it would be worth spending on further research to reduce uncertainty in the cost-effectiveness results, was very small for a number of willingness-to-pay values. Appendix 2 of the ESM displays the results for a willingness to pay of £20,000 per quality-adjusted life-year gained. At this level, the funding decision would be yes for the log-normal and log-logistic models, but no for the remaining models. Despite this, the expected value of information per person was £0 for the gamma, Weibull and Gompertz models, and between £0.09 and £2.04 for the remaining models. This suggests that extrapolation uncertainty is not appropriately captured, as reducing this uncertainty could change the choice of survival model and hence the funding decision. Appendix 2 of the ESM provides further remarks.

Collectively, these results demonstrate that the effects of model choice on uncertainty in both the hazard functions and lifetime mean survival may be substantial, even for models that provide similar point estimates. Hence, analysts could under- or over-estimate the uncertainty in mean survival and hence measures of cost effectiveness unless they carefully consider model selection, in terms of both the model fit during the observed period and quantifying the uncertainty during the extrapolation period.

## Discussion

To our knowledge, this is the first study to examine systematically the properties of seven different commonly used parametric survival models in terms of the uncertainty in estimates of extrapolated hazard and survival functions. We have provided exact analytical expressions for the exponential model and described the use of the delta method and the multivariate normal method for obtaining approximate expressions. Using the four hypothetical datasets, we illustrated how the choice of parametric survival model can strongly affect estimates of uncertainty about the hazard over the extrapolation period, and hence mean survival and cost-effectiveness estimates. For each of the datasets considered, long-term uncertainty in the estimated hazard functions could be constant, increasing or decreasing, depending on the chosen model. We observed substantial differences in the estimated magnitude of uncertainty for estimates of the hazard function, lifetime mean survival and cost-effectiveness estimates.

Our findings are generalisable and applicable to datasets beyond the four used in this study. We have covered a range of commonly observed hazard patterns. Results will be qualitatively the same for other datasets that have similar hazard patterns because of the underlying mathematics that defines the estimated variance in the hazard for a given model. The magnitude of estimates of uncertainty will vary depending on the actual dataset used, but we would expect, for example, that the uncertainty in the hazard of a fitted generalised gamma model may fan out over time whereas that for a log-logistic is likely to narrow over time.

There is existing guidance from the National Institute for Health and Care Excellence Decision Support Unit and in the literature on analysing and extrapolating survival data in cost-effectiveness analyses, which focus on commonly used parametric survival models [2, 17]. This guidance does not discuss the implications of survival model choice on estimates of uncertainty in model functions. A recent discussion on methodological challenges noted that extrapolation involves methodological, structural and parameter uncertainty, and that uncertainty increases as the extrapolated period increases [18]. Our study shows that survival model choice fundamentally influences the estimates of uncertainty in hazard, mean survival and cost effectiveness.

There were some limitations of this work. First, we only examined seven commonly used parametric survival models [2]. There are other models that could be applied, as well as more flexible models such as spline-based models and fractional polynomials [19–22]. Further research into the impact on extrapolation uncertainty of using these models would be beneficial. As noted, six of the seven models that we considered are nested members of the generalised F family [23]. In theory, it may be possible to fit the generalised F model and use significance testing to check if one of the nested models is to be preferred. There are two potential issues with this approach: first, we were not always able to obtain model estimates from the generalised F, secondly, some of the nested models occur as parameters tend to infinity: model testing in this case is not straight-forward [24]. Another limitation is that we did not consider using a piecewise modelling approach, which allows for the data-generating mechanism to be different over time [25]. However, it would not automatically ensure (as might be preferred) that uncertainty increases as the extrapolated horizon increases: this depends on the chosen survival model. Additionally, fitting the extrapolating model to a subset of the sample data leads to a reduced sample size, and estimates of cost effectiveness can be sensitive to the choice of subset [26]. Further, we did not consider a dataset with multiple turning points in the hazard.

In practice, it is important that model choice involves input from clinical experts [2, 27]. This includes understanding both the underlying disease process (data-generating mechanism, or ‘true’ model) and how it evolves over time. The lack of data in the extrapolation period can create uncertainty in the appropriateness of using the fitted model for extrapolation. For example, Davies and colleagues [28] extrapolated survival estimates for two interventions from Weibull models fitted to 8 years of registry data. For one intervention, the model provided accurate predictions for the 8 years, but gave markedly inaccurate predictions when compared with a longer follow-up of the registry data to 16 years. This demonstrates that models that accurately describe the observed data may not provide accurate extrapolations. Hence, it is important to reflect any external evidence (including clinical knowledge) about the possibility that the data-generating mechanism will remain the same in the future. It is likely that there will be uncertainties in any external evidence, thus it is unlikely that their use will fully remove the uncertainties associated with extrapolation.

The results of this study have implications for a health economic analysis. Failure to quantify appropriately uncertainty about inputs, including survival functions, over the observed and extrapolated periods may lead to incorrect estimates of population mean costs and benefits, which may affect reimbursement decisions. As well as affecting estimates of mean cost effectiveness from a probabilistic sensitivity analysis, the choice of survival model will also affect the estimated probability that interventions are cost effective. The results of this study also suggest that the failure to adequately account for extrapolation uncertainty can lead to value of information estimates that are too low.

In Box 1, we outline a set of recommendations for analysts and decision makers who are involved in generating or critiquing extrapolations. These recommendations aim to complement existing guidance (2, 12). We emphasise that considering estimates of uncertainty is important as a component of the extrapolation process.

**Box 1:** Recommendations for analysts and decision makers considering extrapolations from survival models

1. Analysts fitting models to survival data for use in cost-effectiveness models should use input from clinical experts about the underlying disease process over the observed and extrapolated periods. Justification should be provided regarding the implied hazard function, and the assumption that the chosen survival model will be valid for the extrapolation period. Analysts should generate and examine the empirical hazard function to aid in the choice of model based on the sample data.
2. The plausibility of extrapolated hazard estimates is a key part of survival model selection, complementing within-sample goodness of fit. This assessment of plausibility should consider both point and interval (uncertainty) estimates. In general, extrapolations should be associated with uncertainty that increases over time, unless there are compelling arguments to the contrary.
3. In addition to considering the plausibility of extrapolated hazards, the impact on decision uncertainty should also be considered. This may be quantified by the uncertainty in estimates of both lifetime mean survival and cost effectiveness. If follow-up data are almost complete, then differences in estimates of uncertainty in the hazard function are less likely to be of importance.
4. Care should be taken when assuming that a single model for the hazard (and survival) function applies across all time points. Work to consider different models in different time periods should not only consider reflecting the point estimates of the hazard functions, but also consider the implications for uncertainty in these estimates.
5. When reporting results of survival analyses in journal articles or to HTA/reimbursement authorities, a structured analysis of uncertainty should be provided including reporting and visualisation of the uncertainty about hazard functions (as in Fig. 2) and survival functions (as in Fig. 3) and in the mean survival (as in Fig. 4).
6. Analysts and decision makers should use scenario analyses to quantify the sensitivity of estimates of cost effectiveness to survival model choice. If structural uncertainty exists (more than one model structure could be appropriate), then this should be reflected when calculating estimates of uncertainty in the base-case cost-effectiveness results. In the example provided here, this could suggest that the analyst uses the generalised gamma because the uncertainty in its estimates covers almost all of the other competing models (as detailed in Appendix 2 of the ESM).
7. If the use of commonly applied survival models does not adequately reflect individuals’ notions of uncertainty about hazard functions for the extrapolated period, analysts should consider alternative innovative approaches (see the main text for examples).

An important implication for further methodological research is to develop methods on how to incorporate the notion that interval estimates of hazard functions should ‘fan out’ during the extrapolated period. A general approach to characterising extrapolation uncertainty may be required to reflect that we have less knowledge about the data-generating mechanism in the future. A Bayesian approach would provide the ability to both incorporate external information and make probabilistic statements about the parameters of a survival model, taking into account the correlations between these parameters. This external information could include elicited beliefs from clinical experts about survival during the extrapolated period, or the plausibility of different models. Model discrepancy terms can be used to characterise uncertainty in model estimates [29]. An existing case study successfully demonstrated that it is possible to incorporate model discrepancy terms within the extrapolation period with the specific aim of inducing a fanning out of uncertainty in hazard estimates [19]. Further research into this approach should consider how to elicit both discrepancy terms and parameters in survival models [30]. Another advantage of the Bayesian approach is that it removes the need to use a multivariate normal approximation for the joint distribution of parameters in a survival model.

Finally, for this work, we generated representative (hypothetical) datasets, but we did not conduct a simulation study. This was intentional, as the representative datasets were sufficient to highlight the impact of model choice on extrapolation uncertainty. Further research could include a simulation study, to quantify the properties of survival models during the extrapolated period.

## Conclusions

It is important for cost-effectiveness analyses to include realistic estimates of uncertainty about hazard functions and mean survival. This will improve both the accuracy of, and confidence in, reimbursement decisions. The choice of extrapolating model can have a large impact on estimates of uncertainty about hazard functions and lifetime mean survival. As such, consideration of the plausibility of estimates of uncertainty about hazard estimates in addition to point estimates of the hazard, particularly during the extrapolated period, should be informed by clinical knowledge as part of the model selection process. To support this, it is useful to visualise the observed and modelled hazard estimates as shown in the case study examples in this article. We provide seven new and specific recommendations for analysts and decision makers to follow when considering the uncertainty in the extrapolated period and the impact of parametric survival model choice.

## Electronic supplementary material

Below is the link to the electronic supplementary material.

Supplementary material 1 (DOCX 18 kb)

## Data Availability

The four representative datasets and the analysis code used in this study are available at: https://figshare.shef.ac.uk/articles/Data_Rda/9751907/2 and https://figshare.shef.ac.uk/articles/Analysis_R/9751913/2.
