# Supplementary material for: How Uncertain is the Survival Extrapolation? A Study of the Impact of Different Parametric Survival Models on Extrapolated Uncertainty About Hazard Functions, Lifetime Mean Survival and Cost Effectiveness
Source: Pharmacoeconomics. 2019 Nov 25;38(2):193–204. doi: 10.1007/s40273-019-00853-x (PMC6976548; doi:10.1007/s40273-019-00853-x)
Supplement: Supplementary file 1 — Supplementary material 1 (DOCX 18 kb) [file 40273_2019_853_MOESM1_ESM.docx]

## Appendix 1: Applying the delta method to estimate the variance of the hazard for the Weibull and exponential survival models.

Without loss of generality, let $\theta$ denote the model parameters, and $g\left( \theta\right)$ denote a function of these. Then the variance of $g\left( \theta\right)$ is approximated as:

$Var\left( g\left( \theta\right) \right)\approx\left( \frac{dg\left( \theta\right)}{d\theta} \right)^{2}var\left( \theta\right)$ (Eq A1)

For example, in the case of an exponential model, the hazard function $g\left( \theta\right)= \lambda$, and its derivative $=1$. Hence, for the hazard function of an exponential model, Equation (A1) reduces to Equation (3).

When there are multiple parameters $\theta=\left( \theta_{1},\ldots,\theta_{k} \right)$, equation (6) may be expanded to:

$Var\left( g\left( \hat{\theta} \right) \right)\approx\sum_{i=1}^{k} Var\left( \theta_{i} \right)\left( \frac{\partial f}{\partial\theta_{i}} \right)^{2}+2\sum_{i<j}^{k} \sum Cov\left( \theta_{i},\theta_{j} \right)\left( \frac{\partial f}{\partial\theta_{i}} \right)\left( \frac{\partial f}{\partial\theta_{j}} \right)$ (Eq A2)

For example, in the case of a Weibull model with scale parameter, $\lambda$, and shape parameter, $\gamma$, and $\theta=\left( \lambda,\gamma\right)$, the delta method approximation of the variance of the estimated hazard function is:

$$Var\left( g\left( \hat{\theta} \right) \right)\approx\left( \frac{d\left( \hat{\lambda}\hat{\gamma}t^{\hat{\gamma}-1} \right)}{d\hat{\lambda}} \right)^{2}Var\left( \hat{\lambda} \right)+\left( \frac{d\left( \hat{\lambda}\hat{\gamma}t^{\hat{\gamma}-1} \right)}{d\hat{\gamma}} \right)^{2}Var\left( \hat{\gamma} \right)+2\left( \frac{d\left( \hat{\lambda}\hat{\gamma}t^{\hat{\gamma}-1} \right)}{d\hat{\lambda}} \right)\left( \frac{d\left( \hat{\lambda}\hat{\gamma}t^{\hat{\gamma}-1} \right)}{d\hat{\gamma}} \right)Cov\left( \hat{\lambda,}\hat{\gamma} \right)$$

$$=\left( \hat{\gamma}t^{\hat{\gamma}-1} \right)^{2}Var\left( \hat{\lambda} \right)+\left( \hat{\lambda}\hat{\gamma}t^{\hat{\gamma}-1}\log\left( t \right)+t^{\hat{\gamma}-1}\hat{\lambda} \right)^{2}Var(\hat{\gamma})$$

 $+2\left( \hat{\gamma}t^{\hat{\gamma}-1} \right)\left( \hat{\lambda}\hat{\gamma}t^{\hat{\gamma}-1}\log\left( t \right)+t^{\hat{\gamma}-1}\hat{\lambda} \right)Cov\left( \hat{\lambda},\hat{\gamma} \right),$ (Eq A3).

It is clear that Equation (A3) is a function of $t^{\hat{\gamma}-1}$ and $\text{log}\left( t \right)$. Hence, if $\hat{\gamma}>1$ the variance of the hazard function will increase over time, whereas if $\hat{\gamma}<1$ then the term $t^{\hat{\gamma}-1}$ will decrease over time, although it may be possible for the uncertainty to increase over time, depending on how the rate of decrease of $t^{\hat{\gamma}-1}$ compares to the rate of increase of $\log\left( t \right)$ over time.

## Appendix 2: Summary cost-effectiveness results

For the six candidate models (the exponential provided a visually poor fit to the observed data, so was not considered further) the probabilistic mean incremental cost-effectiveness ratio and its percentile-based 95% confidence interval are displayed in Table A1. Models are ordered by the width of the confidence interval. Of the six candidate models, three (Weibull, Gompertz, generalised gamma) assumed that the observed increasing hazard continued during the extrapolated phase. The other three models (gamma, lognormal and log-logistic) assumed that the extrapolated was either (roughly) constant or decreasing. These alternative assumptions can be viewed as representing the competing aspects of ageing, which increases hazards, and frailty, which decreases hazards. Note that the generalised F model is not included. Under the default flexsurv settings parameter estimation finished without any reported problems. However, all point estimates from 1.25 years onwards were non-finite, with 95% confidence intervals of approximately 0.03 to 30. This suggested that extrapolations from the generalised F model were not reliable, and so they were not used to perform a cost-effectiveness analysis.

The variation in hazard estimates observed in Figure 2 is reflected by the variation in incremental cost-effectiveness ratio ( per quality adjusted life year gained) estimates; from approximately £18,000 (log-logistic and lognormal models) to £35,000 (Gompertz model). If it were not possible to choose between the plausibility of the competing models, then preference would be given to the generalised gamma as it is more likely to represent extrapolation uncertainty, having the widest confidence intervals about the incremental cost-effectiveness ratio estimate. Of note, based on point estimates, it would not be possible to choose between the Weibull and generalised gamma models. Both provide almost identical mean estimates (£29,600 and £29,500, respectively). Given that the future is associated with substantial uncertainty, a default approach would be to choose the generalised gamma, with its wider confidence interval, unless there are compelling reasons to assume that there is little extrapolation uncertainty. Finally, it is noted that the 95% confidence interval from the generalised gamma includes the point estimates from three of the other five models. None of the confidence intervals from the remaining models includes more than one other model.

Table A1: Summary cost-effectiveness results.

| **Model** | | **Mean incremental cost*** | **Lower 95% CI** | **Upper 95% CI** | **CI Width** | **Expected value of perfect information**** |  |
| --- | --- | --- | --- | --- | --- | --- | --- |
| Lognormal | | £18,458 | £16,469 | £20,895 | £4,426 | £2.04 |  |
| Log-logistic | | £17,714 | £15,799 | £20,551 | £4,752 | £0.54 |  |
| Gamma | | £23,315 | £20,785 | £25,939 | £5,154 | £0 |  |
| Weibull | | £29,476 | £25,840 | £34,200 | £8,360 | £0 |  |
| Gompertz | | £35,277 | £30,211 | £40,657 | £10,446 | £0 |  |
| Generalised gamma | | £29,557 | £21,815 | £36,320 | £14,506 | £0.09 |  |
|  | CI: Confidence interval *per quality adjusted life year gained **per person at willingness to pay of £20,000. | | | | | | |
